# Exploring protein structural ensembles: Integration of sparse experimental data from electron paramagnetic resonance spectroscopy with molecular modeling methods

**DOI:** 10.7554/eLife.99770

**Published:** 2024-09-16

**Authors:** Julia Belyaeva, Matthias Elgeti

**Affiliations:** 1 Institute for Drug Discovery, Leipzig University Medical School Leipzig Germany; 2 Institute for Medical Physics and Biophysics, Leipzig University Medical School Leipzig Germany; 3 Integrative Center for Bioinformatics, Leipzig University Leipzig Germany; University College London United Kingdom; Boston University United States

**Keywords:** EPR spectroscopy, molecular modeling, computational structural biology, integrative structural biology, protein dynamics, DEER

## Abstract

Under physiological conditions, proteins continuously undergo structural fluctuations on different timescales. Some conformations are only sparsely populated, but still play a key role in protein function. Thus, meaningful structure–function frameworks must include structural ensembles rather than only the most populated protein conformations. To detail protein plasticity, modern structural biology combines complementary experimental and computational approaches. In this review, we survey available computational approaches that integrate sparse experimental data from electron paramagnetic resonance spectroscopy with molecular modeling techniques to derive all-atom structural models of rare protein conformations. We also propose strategies to increase the reliability and improve efficiency using deep learning approaches, thus advancing the field of integrative structural biology.

## Introduction

### The conformational landscape and its role for protein function

**Figure 1.**
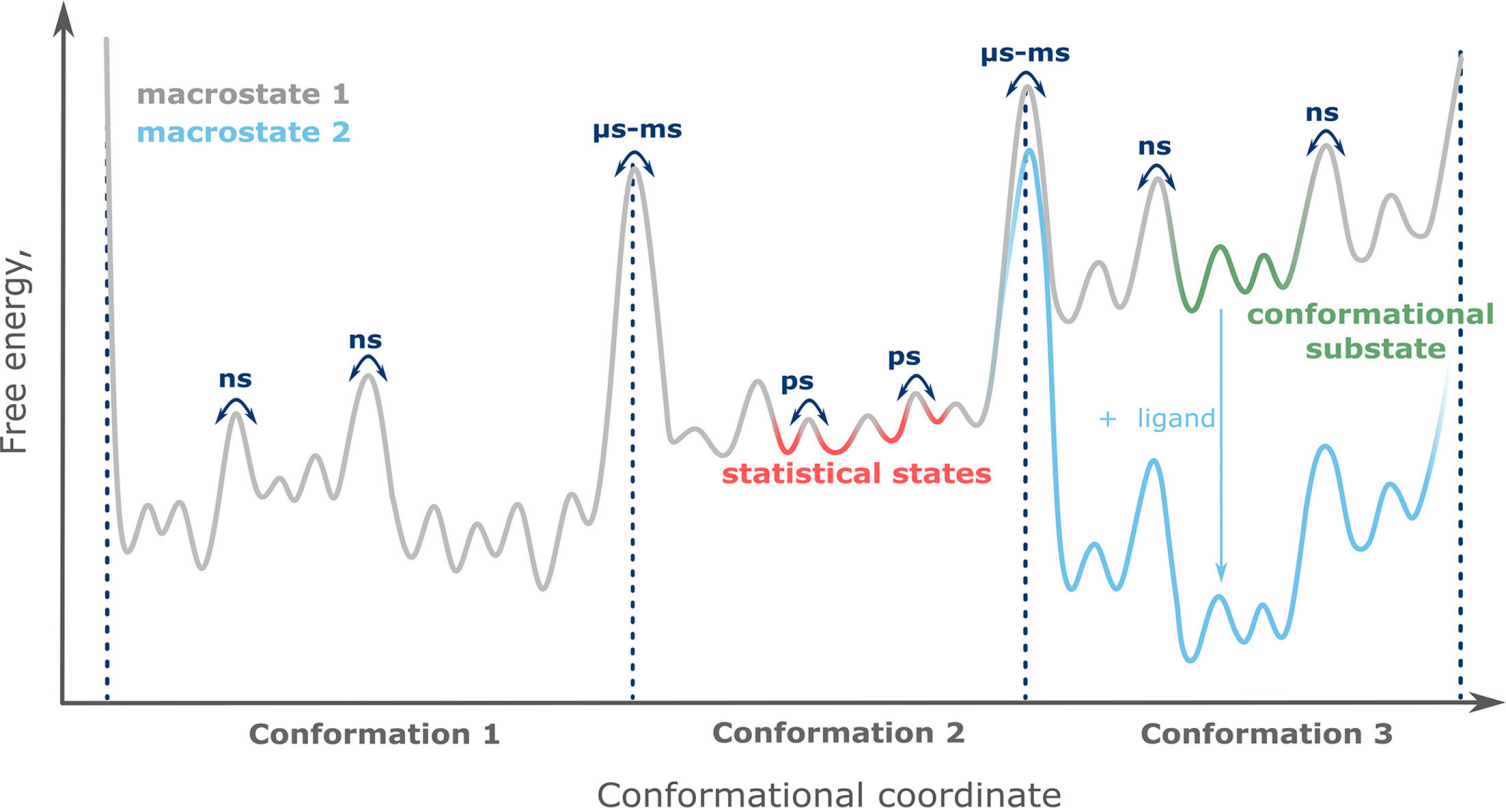
The conformational landscape of proteins.

Under physiological conditions, most proteins are highly dynamic, adopting various structures with distinct probabilities. The diversity and thermodynamics of protein structures may be conceptualized as a *conformational landscape*, which represents a low-dimensional projection of the multidimensional free energy surface of generalized protein coordinates (Figure 1). Following the nomenclature introduced by Frauenfelder et al., a *macrostate* represents the global thermodynamic state of a protein defined by the physical and (bio)chemical conditions, such as temperature, pressure, chemical potential, type, and concentration of solutes or ligands (Frauenfelder et al., 1991). Within a given macrostate, protein structural rearrangements may occur on different timescales. For example, *conformational states* (or simply *conformations*) are separated by barriers of several kT and thus interconvert on the timescale of microseconds to milliseconds. Within each conformational state, fluctuations which occur on the order of nanoseconds separate individual conformational substates, while even faster transitions occur between statistical substates (Frauenfelder et al., 1991; Henzler-Wildman and Kern, 2007). It is conceivable that the macrostate defines the equilibrium distributions of the entire *conformational ensemble* comprising all timescales (Shi et al., 2015).

Each conformational state contributes a specific functional profile. Thus, protein function is defined by the distribution of conformational states, and their redistribution upon interaction with binding partners such as ligands (Figure 1). This functional framework entails that even sparsely populated conformational states can rise to functional relevance and thus should be considered when a protein is targeted pharmacologically. This was recently demonstrated for the most prominent pharmacological targets, G-protein-coupled receptors (GPCRs), where minute amounts of active conformation lead to basal receptor activity (Lerch et al., 2020).

The three tiers of protein states are defined by their timescales of interconversion. Conformational states exchange on the slow, micro- to millisecond timescale (dotted lines), while conformational substates (green) and statistical substates (red) have lifetimes of nanoseconds or picoseconds, respectively. Changing the protein’s macrostate, for example by adding ligand (blue), leads to redistribution of the conformational equilibrium.

While various computational methods have been developed for the characterization of conformational landscapes, slow timescales (milliseconds and beyond) are still challenging to access. These shortcomings can be addressed by using complementary experimental methods, which provide access to slow conformational exchange and can resolve the equilibrium ensemble under (near-) physiological conditions. The integration of these experimental results with molecular modeling culminates in high-resolution structures of rare conformational states and their thermodynamics (Allison, 2017).

### Experimental methods for studying conformational landscapes

Currently, the most commonly used approaches to study protein structure include X-ray diffraction, cryo-electron microscopy (EM), nuclear magnetic resonance (NMR) spectroscopy, Förster resonance energy transfer (FRET), and electron paramagnetic resonance (EPR) spectroscopy. Each of these methods has its strengths and limitations. For instance, structures determined by X-ray diffraction provide high resolution, however, the conformational state most stable under crystal conditions may lack physiological relevance (Freed et al., 2010; Dasgupta et al., 1997; Rasmussen et al., 2011). Furthermore, the requirements of crystallization narrow the applicability of X-ray crystallography, because many proteins exhibit flexible regions which prevent crystallization or diminish resolution. This is especially true for membrane proteins, where shortening of dynamics loops or insertion of highly soluble and rigid proteins circumvent this problem (Carpenter et al., 2008; Lacapère et al., 2007; Ding et al., 2021; Thorsen et al., 2014). In order to access conformational dynamics, X-ray structural models commonly serve as a starting point for molecular modeling, for example using molecular dynamics (MD) simulations. However, slow conformational changes (>10^−5^ s) remain challenging to follow with atomic resolution.

Cryo-EM may in principle directly explore conformational ensembles, and rapid vitrification enables the characterization of macrostates (Glaeser, 2016; Kühlbrandt, 2014; Yip et al., 2020). Though freezing may still introduce bias toward specific conformations, cryo-EM is the most rapidly advancing method for investigating conformational ensembles of proteins (Twomey et al., 2015; Mehra et al., 2020; Bonilla and Kieft, 2022; Noble et al., 2018; Karplus and McCammon, 2002). Pairing individual structural models from cryo-EM with MD simulations has recently given valuable insight into the activation process of G proteins by an activated GPCR (Papasergi-Scott et al., 2023). However, flexible membrane proteins may adopt a vast number of conformational states constituting a great obstacle to current classification approaches.

Most commonly used NMR methods effectively explore structural and dynamic features of small- and medium-sized proteins, typically up to 300 amino acid residues (Clore and Gronenborn, 1991; Opella and Marassi, 2004; Kaptein and Wagner, 2015). Site-specific fluorine NMR extends these capabilities to larger proteins, also enabling observation of dynamic events spanning from nanoseconds to seconds (Prosser and Alonzi, 2023; Danielson and Falke, 1996). Computational integration of NMR data remains challenging due to the added difficulty of assigning resonances to specific residues. Novel, detector-based methods elegantly integrate NMR timescale information with computational modeling (Smith et al., 2023), but access to large amounts of functional sample as well as acquisition and processing times remain limiting factors.

FRET detects conformational changes in the range of 30–80 Å and, in combination with single particle analysis (smFRET), provides access to conformational heterogeneity and exchange dynamics (Ha, 2001; Agam et al., 2023; Gregorio et al., 2017; Zhao et al., 2024). However, fluorophores represent relatively large, flexible, and hydrophobic probes, which limits spatial resolution or may disturb the local structure and dynamics (Sánchez-Rico et al., 2017; Peter et al., 2022).

In this review, we focus on EPR spectroscopy, which allows the investigation of protein dynamics across a broad range of timescales (from picoseconds to seconds or longer) with few restrictions on sample conditions. The application of pulse EPR spectroscopy adds further capabilities in terms of spatial resolution and accurate quantification of individual conformational states. A continuously growing number of computational tools are becoming available assisting with the integration of EPR spectroscopic data and providing a detailed picture of structural dynamics underlying protein function.

### Site-directed spin labeling EPR spectroscopy

EPR spectroscopy comprises a large toolbox of methods enabling the exploration of protein systems containing paramagnetic centers. Since unpaired electrons are usually depleted during protein expression, stable radicals need to be introduced for example via site-directed spin labeling in order to obtain an EPR signal (Torricella et al., 2021; Pierro and Drescher, 2023; Jana et al., 2023). Several continuous wave (CW) EPR methods have been developed to study the different timescales of protein dynamics, and gain insight into structure and population of conformational states within an ensemble (Table 1). In the following, we limit our considerations to studies with nitroxides, which are by far the most commonly used spin labels. However, especially for distance measurements several other spin label side chains have been developed, each exhibiting benefits and drawbacks compared to nitroxides that are discussed elsewhere (Fielding et al., 2014). The *CW EPR lineshape* (first derivative of the absorption spectrum) is highly sensitive to spin label dynamics on the 100 ps to 100 ns timescale which is strongly influenced by structure and dynamics of the protein (Hubbell et al., 1996; Campbell et al., 2022; Mchaourab et al., 1996; Fichou et al., 2019; Pierro et al., 2020). Coexistence of several conformational states leads to superimposed, complex EPR spectra. While a comprehensive theory of spin label motion exists, the interpretation of CW EPR lineshapes remains challenging due to a large number of parameters. In particular cases, when the selection of fitting parameters during lineshape analysis is ambiguous, statistical analysis becomes necessary to assess the likelihood of one parameter set over another (Francis et al., 2012; Etienne et al., 2023; Lindemann et al., 2020).

**Table 1.** Summarizing information on electron paramagnetic resonance (EPR) spectroscopic techniques.

Method	Features				References
	Dynamics (timescale)	Structure(resolution)	Population	Computational analysis	
CW EPR	Yes (10^−10^ to 10^−7^s)	Yes, via scanning (topology)	Yes, ≤3 conformations	Semi-empirical and lineshape analysis	Marsh, 1981; Hubbell and Altenbach, 1994; Columbus and Hubbell, 2002
ST EPR	Yes (10^−7^ to 10^−3^ s)	No	No	Heuristic analysis	Hyde and Dalton, 1972
TR EPR	Yes (>10^−3^ s)	No	Yes	Lineshape analysis	Farahbakhsh et al., 1993
DEER	No	Yes (<10^−10^ m)	Yes	Parametric and non-parametric fitting models	Jeschke, 2012
ENDOR	No	Yes (>10^−11^ m)	Yes	Lineshape analysis	Lubitz et al., 2002
SR EPR	Yes, (10^−6^ to 10^−5^ s)	No	Yes, ≤2 conformations	Exponential fitting	Bridges et al., 2010

Further information on protein topology can be obtained via power saturation CW EPR spectroscopy, which is often combined with spin label scanning. Here, spin labels are introduced to successive sites along a sequence of amino acids and the influence of paramagnetic substances on the saturation behavior is evaluated (Altenbach et al., 1990; Hubbell et al., 2003). In general, full seqeunce coverage with spin labels is desired to uncover even subtle changes in structure and dynamics of the protein segment of interest. However, evaluating or comparing specific secondary structure models with different periodicities, such as α-helix or β-sheet, requires only a strongly reduced set of spin labeling sites. Saturation transfer (ST) EPR and time-resolved (TR) EPR represent two other methods utilizing continuous microwave radiation, extending sensitivity to the timescales of microseconds to milliseconds (Hyde and Dalton, 1972; Hyde and Thomas, 1973; Schwarz et al., 1990; Rayes et al., 2011) and millisecond to hours (Steinhoff et al., 1994; Farahbakhsh et al., 1993; Knierim et al., 2007), respectively. While several approaches for the analysis of ST EPR spectra have been developed (Hustedt and Beth, 2004), these represent purely heuristic methods and will therefore not be discussed in more detail. The dynamic processes picked up by TR EPR are too slow to be modeled using all-atom modeling techniques and are also outside the focus of this review. Notably, all EPR methods mentioned so far exhibit little to no restrictions on the experimental conditions, including a wide range of temperatures or different environments (solution, membranes, living cells, etc.).

The power of EPR spectroscopy is strongly expanded by the application of microwave pulses (pulse EPR spectroscopy). Four-pulse double electron–electron resonance energy transfer (DEER), also known as pulsed electron–electron double resonance (PELDOR), is a pulsed EPR spectroscopic technique usually performed on frozen solutions, in order to resolve distances between two spin labels at sub-Angstrom resolution. This method captures interspin distances ranging from 1.5 to 8.0 nm, and even up to 16.0 nm in fully deuterated samples (Peter et al., 2022; Jeschke, 2012). Moreover, DEER experiments elegantly connect structure and thermodynamics of proteins by resolving the conformational ensemble in probability distance distributions (Elgeti and Hubbell, 2021; Evans et al., 2020; Dawidowski and Cafiso, 2013; Wingler et al., 2019). This makes DEER the prime method for computational integration with structural biology which will be discussed in detail. Electron-nuclear double resonance (ENDOR) assesses hyperfine interactions among magnetic nuclei and paramagnetic centers within solute samples cooled to cryogenic temperatures. It can be implemented as both CW and pulse technique (Lubitz et al., 2002; Weber et al., 2001). ENDOR is effective for revealing the structures of specific parts of protein molecules, achieving atomic-level accuracy in distances below 1.5 nm (Lendzian et al., 1996). Recent work has demonstrated that using fluorinated amino acids (19F-ENDOR), in particular in combination with Gadolinium spin labels, extends the upper distance limit to above 2 nm (Bogdanov et al., 2024). Several computational methods have been developed to simulate ENDOR spectra, however, integration with structural models has not been achieved yet (Meyer et al., 2020; Meyer et al., 2022; Stoll and Schweiger, 2006). Lastly, saturation recovery EPR (SR EPR) represents another pulsed EPR technique used to gain insights into protein dynamics. It enables the resolution of dynamic events on the low to intermediate microsecond timescale which remains difficult to access with other methods (Bridges et al., 2010; Sarewicz et al., 2008; Yang et al., 2015). However, so far no computational approaches for the structural integration of SR EPR have been developed.

### Analysis and interpretation of experimental CW EPR data

The sensitivity of CW EPR to molecular motion arises from the incomplete averaging of anisotropic magnetic interactions leading to characteristic lineshapes.

#### Semi-empirical analysis methods

**Figure 2.**
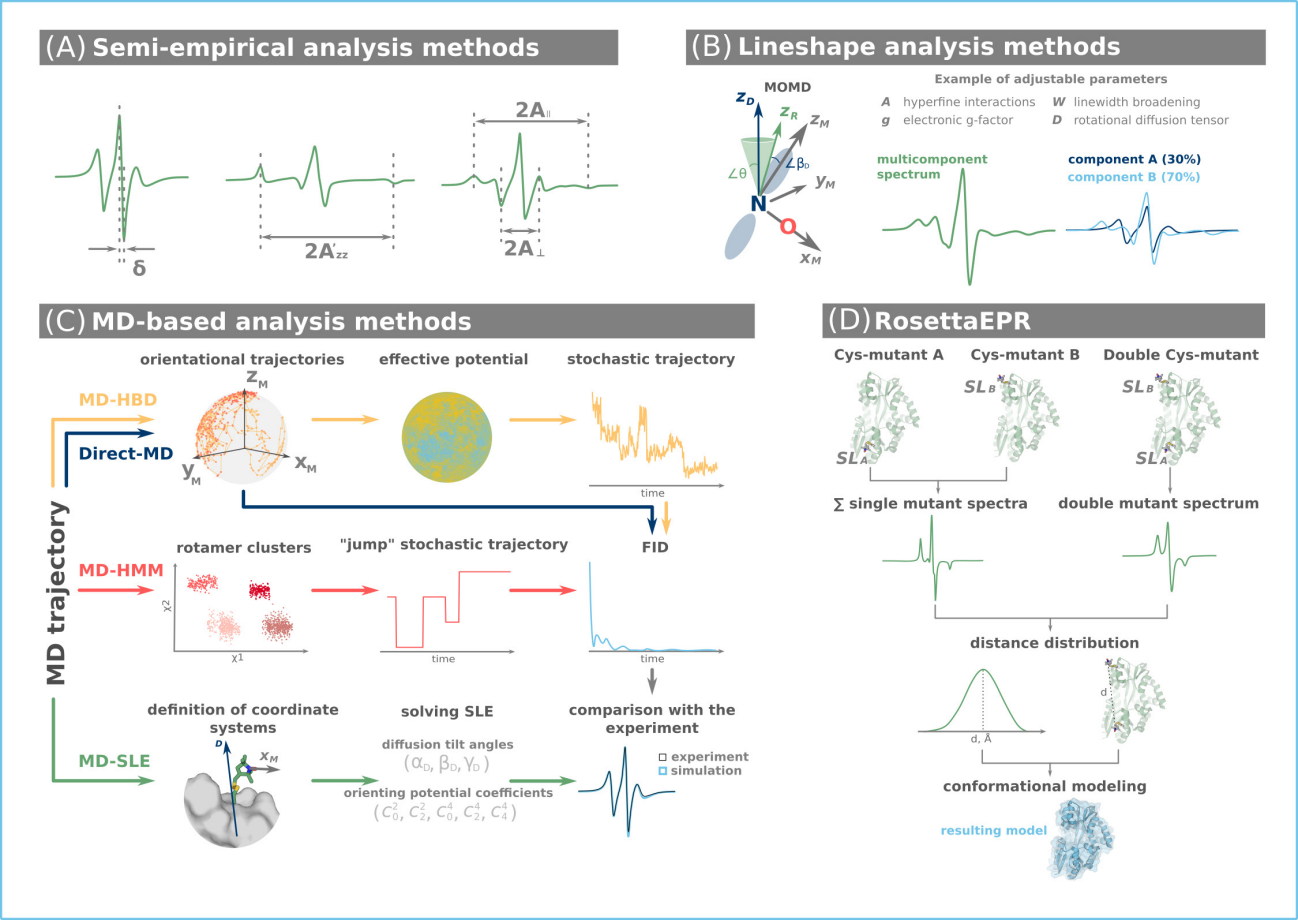
Computational approaches for the analysis and interpretations of continuous wave (CW) electron paramagnetic resonance (EPR) data. (**A**) Semi-empirical analysis of the CW EPR lineshape provides insight into the rate of motion (*δ*), polarity (*A*’_*zz*_) of the spin label environment, parallel (*A*_॥_), and perpendicular (*A*_⊥_) compounds of CW EPR spectra of spin-labeled membrane bilayer lipids. (**B**) Lineshape analysis provides access to motional parameters and populations of individual equilibrium conformations. (**C**) Molecular dynamics (MD) simulations may explore the entire conformational landscape of a protein and provide data to simulate the CW EPR spectrum. The latter is then compared with the experiment. (**D**) RosettaEPR approach uses results from single and double mutant CW EPR experiments to derive distance constraints for subsequent conformational modeling.

Derive parameters of molecular motion directly from the lineshape. Columbus and Hubbell showed that the distance between the minimum and maximum of the CW EPR lineshape, the center linewidth *δ* (Figure 2A), is strongly related with the correlation time and order parameter of the observed motion (Columbus and Hubbell, 2002). Also, the effective hyperfine splitting *A*’_*zz*_, as assessed by the distance between the outer minima can be used to determine the correlation time or the polarity of spin label environment (Freed, 1976; Altenbach and Hubbell, 2015). Axially symmetric systems such as spin-labeled lipids of a membrane bilayer can be analyzed using the parallel (*A*∥) and perpendicular (*A*⟂), which significantly simplifies the analysis of the experimental results (Subczynski et al., 2010).

#### Lineshape analysis

Motional models for analyzing CW EPR lineshapes computationally exist at varying degrees of complexity. Most widely used models for the description of intermediate to slow spin label dynamics solve the stochastic Liouville equation (SLE) and assume microscopic order and macroscopic disorder (MOMD, Figure 2B; Meirovitch et al., 1984; Budil et al., 2006). Such models can include ordering potentials in the lineshape analysis to characterize the amplitude of molecular motions. However, while intricate motional models can be implemented, distinct parameter sets may result in equally good mathematical fits. To this end, strongly correlated fitting parameters indicate that the complexity of the model (i.e., number of parameters) should be reduced to avoid overfitting (Altenbach and Budil, 2024). Motion faster than 1 ns leads to complete averaging of anisotropic magnetic interactions. This simplifies the analysis and an effective Hamiltonian can be used (Hubbell and McConnell, 1971).

Analysis via lineshape simulation includes iterative adjustment of model parameters to fit the experimental spectra. Such methods are implemented in the programs EasySpin, NLSL, MultiComponent, Spinach, Simlabel, and cwepr (Stoll and Schweiger, 2006; Altenbach and Budil, 2024; Budil et al., 1996; Altenbach and Hubbell, 2024; Schröder and Biskup, 2022). These software toolkits require relatively low computational resources and have a user-friendly graphical interface. Lineshape simulations offer valuable insights into the protein local structure, dynamics, topology, conformational changes, and interactions between binding partners. Notably, when a protein conformational change affects spin label dynamics sufficiently leading distinct lineshapes, lineshape analysis can disentangle such multicomponent spectra (Figure 2B) and thus describe conformational equilibria (Altenbach and Budil, 2024). However, CW EPR spectra represent a convolution of spin label and protein dynamics (Mchaourab et al., 1996) and when spin label and protein motions occur on similar timescales, lineshape analysis is reaching its limits. In such cases, integrative analysis approaches incorporating molecular modeling will provide a possible alternative.

#### MD-based approaches for CW EPR analysis

The majority of integrative methods for the analysis of CW EPR data rely on MD simulations of spin-labeled proteins. *MD-SLE* (Figure 2C) approach combines short MD simulations with solving the SLE to analyze CW EPR data (Budil et al., 2006; Stoica, 2004). Short MD trajectories corrected for translation and rotation of the protein are assumed to describe only spin label motion. Such trajectories serve as inputs for SLE-solving lineshape analysis. Combining MD-SLE with high-field CW EPR experiments, where protein motions are assumed frozen, further validates the separate treatment of protein and spin label dynamics (Barnes et al., 1999).

Two other approaches construct simplified models of the spin labels dynamics from short MD trajectories, namely hindered Brownian dynamics (*MD-HBD*, Figure 2C) and hidden Markov models (*MD-HMM*). These methods generate long-scale stochastic trajectories of spin label dynamics. Stochastic trajectories are then used to compute the trajectory of magnetization, also known as free-induction decay (FID). From the FID, the CW EPR spectrum is reconstructed via Fourier transform (Martin et al., 2019; Steinhoff and Hubbell, 1996; Beier and Steinhoff, 2006; White et al., 2007; Sezer et al., 2008a; Sezer et al., 2009). Besides MD-HBD and MD-HMM, there is also the *Direct-MD* approach (Figure 2C), which is becoming increasingly popular. It models the magnetization trajectory from longer MD trajectories of spin-labeled proteins without employing stochastic modeling of spin label dynamics (Martin et al., 2019; Oganesyan, 2011; Tyrrell and Oganesyan, 2013).

MD-HBD and MD-HMM have proven effective for calculating the CW EPR spectrum of small- and medium-sized proteins that adopt a single conformational state. However, modeling multiple conformations of large- and medium-sized proteins requires extensive MD simulations lasting several microseconds or longer. While this was still challenging a few years ago, the advent of GPU-based computing made such simulations available to a larger community. We suggest that MD-based approaches, which effectively sample the conformational states of spin labels, should be integrated with deep learning techniques, such as AlphaFold2, RoseTTAFold, or ESMFold (Jumper et al., 2021; Baek et al., 2021; Krishna et al., 2024; Jeliazkov et al., 2023), to enhance conformational sampling of proteins. Structural models of protein conformations produced by such neural networks should be validated and refined through replicas of MD simulations including spin labels and by comparison with experiments (Baek et al., 2021; Sala et al., 2023). To this end, the benefit of combining MD simulations with deep learning is twofold: providing experimentally validated all-atom structural information while assisting with the interpretation of complex CW EPR spectra.

Rosetta is a software toolbox with a wide range of applications, including molecular design, folding, docking, and modeling tools (Leman et al., 2020). Rosetta uses a library of protein fragments and employs Monte-Carlo assembly to construct structural models of protein conformations. These resulting models are then evaluated with a physics-based scoring function. RosettaNMR represents the first algorithms for the de novo prediction of protein structures, which integrated experimental NMR data. This was the basis for the development of the RosettaEPR. *RosettaEPR* integrates interspin distances into the modeling process, which are derived from exchange broadening via CW EPR of doubly spin-labeled protein (Alexander et al., 2008). The distance range accessible using this approach is limited to <~25 Å. Thus, it should be noted that each line-broadening analysis requires three CW EPR experiments. One experiment with the doubly spin-labeled mutant, and two experiments with each individual single mutant of the spin pair (Farrens et al., 1996; Rabenstein and Shin, 1995; Altenbach et al., 2001). In summary, RosettaEPR represents a computationally efficient approach for conformational modeling, which includes experimental data and does not require a structural template of the protein. However, in contrast to MD approaches, Rosetta does not allow for the observation of time-dependent conformational changes.

### Integration of distance information derived from experimental DEER data

Pulsed dipolar spectroscopy (PDS) in combination with site-directed spin labeling (SDSL) gives access to distance distributions between two coupled spins. While several different PDS pulse sequences exist, the most commonly used method is 4-pulse deadtime-free DEER, for which recently application guidelines have been put forward (Pannier et al., 2000; Schiemann et al., 2021). Experimental DEER data consist of time-dependent spin echo intensities (dipolar evolution), which can be translated into interspin distance distributions. In addition to the dipolar interaction of intramolecular spins, DEER signals also contain intermolecular contributions (background), which must either be included in the analysis or subtracted a priori. Several different analysis methods have been developed, which are concisely reviewed in the following. A more detailed introduction including benchmark tests can be found elsewhere (Russell et al., 2022).

#### Model-free analysis

Model-free analysis of DEER data presents a mathematically ill-posed problem that is typically addressed by Tikhonov regularization which essentially smooths the distance distribution. Adequate smoothness is typically chosen via the L-curve criterion of the regularization parameter, but other methods such as the Akaike information criterion corrected or the Bayesian information criterion exist, and determine the level of detail observed in the analyzed distance distributions (Edwards and Stoll, 2018). While a minimum width of distance peaks makes physical sense, taking into account the conformational entropy of the labels and the protein, the assumption of equal widths for all distance peaks is inconsistent with the heterogeneous picture of a conformational state (Figure 1). The evaluation of populations, one of the main virtues of DEER, is complicated because it requires a posteriori fitting of the distance distribution to a linear combination of parametric distributions with quantifiable area, such as Gaussians.

#### Model-based analyses

Model-based analyses, such as Gaussian mixture models, assume that DEER distributions represent a superposition of individual distance peaks. Each peak has a specific shape that is described by parameters such as mean position, peak width, and amplitude. This approach dramatically reduces the number of fitting parameters during analysis and provides direct access to populations of individual peaks as well as confidence intervals of each fitting parameter. In addition, simultaneous (global) analysis of multiple DEER datasets of the same spin pair recorded under different conditions further increases the confidence in parameter values such as peak positions, populations, or background parameters (Hustedt et al., 2021; Jeschke et al., 2006; Khan et al., 2023).

Both Tikhonov regularization and parametric models are included in widely used software packages, such as the Python toolbox DEERlab or DeerAnalysis (Fábregas Ibáñez et al., 2020; Stein et al., 2015). More recently, DEER analysis methods using *deep learning* have been developed. These tools include neural networks trained on a large dataset of synthetic DEER data (Worswick et al., 2018) and show comparable or even improved reliability (Casiraghi et al., 2024). The most prominent example is DeerNet, which is included in the DeerAnalysis2022 and Spinach software packages (ETH Zurich, 2023; Hogben et al., 2011).

One main challenge in interpreting DEER results is peak assignment. In principle, each distance peak is due to a specific protein and label conformation. Thus, if a peak appears shifted, for example under altered ligand conditions, the origin of this shift could be due to a change of the protein or label conformation, or a combination of both. One way to tackle this problem is to select surface-exposed spin labeling sites. This way, when the protein changes conformation, the ensemble of spin label rotamers remains unaffected. Surface exposure can be verified using CW EPR as the lineshape provides a sensitive monitor of spin label dynamics. Once this condition is met, the DEER analysis can be reduced to conformational states and populations of the protein, thus directly linking protein structure and thermodynamics.

PDS methods such as DEER depend on two paramagnetic centers being in proximity, and thus can be used to evaluate and characterize protein oligomerization. Studies evaluating the monomer/dimer equilibrium, the dimer architecture, and functionality have been conducted all of which utilizing distance information and modulation depth parameters obtained from DEER experiments (Hilger et al., 2005; Bergdoll et al., 2018; Pliotas et al., 2012). In cases when more than two spins are present per nano-object, data analysis needs to be amended by power scaling to avoid artificial ‘ghost distances’ (Evans et al., 2020; von Hagens et al., 2013; Khan et al., 2023).

Notably, membrane proteins are commonly investigated in a detergent solubilized form to prevent oligomer formation. Generally, the different properties of detergent and lipid molecules lead to altered spin label dynamics which are easily picked up by CW EPR (Flores Jiménez et al., 2011). Interestingly, the changed label dynamics do not lead to dramatic structural alterations and the DEER distances in different systems are often quite similar. Obviously, this is not necessarily true for the position of conformational equilibria, which are often sensitive to the environmental parameters such as lipid or detergent composition (Van Eps et al., 2017).

### Methods for simultaneous modeling of protein and spin label dynamics

Several molecular modeling techniques have been developed to simulate the dynamics of proteins and spin labels. They provide atomistic models of protein conformations that can explain sparse DEER experimental data. Molecular modeling approaches simulate protein and spin labels either simultaneously (combining approaches) or separately (discriminating approaches).

**Figure 3.**
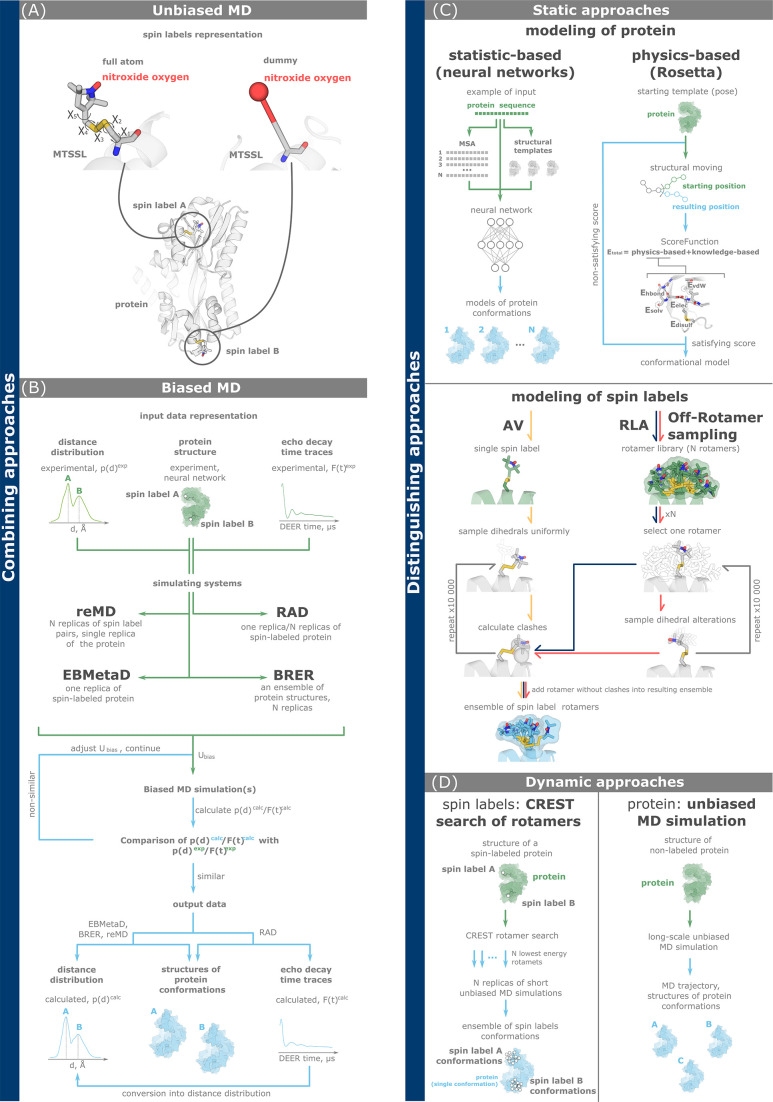
Computational methods for double electron–electron resonance (DEER) data analysis and integration. (**A, B**) Combining approaches simultaneously model the dynamics of both a protein and spin labels. Full-atom and dummy representations of spin labels are possible. (**C, D**) Discriminating approaches investigate conformations of a protein and spin labels separately. (**A**) Unbiased molecular dynamics (MD) simulations of a spin-labeled protein. (**B**) Biased MD approaches add biasing potential to the simulating system according to the principle of maximum entropy. The potential is gradually adjusted based on the degree of agreement between simulated and experimental data, including distance distributions (reMD, EBMetaD, and BRER) and echo decay time traces (restrained average dynamics, RAD). (**C**) Static approaches explore the conformations of either proteins (top) using statistical and physics-based methods, or spin labels (bottom) using accessible volume (AV, yellow arrows), rotamer library approach (RLA, dark blue arrows), and off-rotamer sampling (red arrows). (**D**) Dynamic discriminating approaches use MD-based techniques to investigate the conformational landscapes of either spin labels (left, CREST/MD) or a protein (right, unbiased MD). In the latter case, both full-atom and coarse-grained representations of the protein are possible.

Most *combining approaches* (Figure 3B), such as restrained-ensemble MD (*reMD*), ensemble-biased metadynamics (*EBMetaD*), and bias-resampling ensemble refinement (*BRER*), steer MD simulations of spin-labeled proteins toward a conformational ensemble that accurately reproduces the experimental interspin distance distributions. This is achieved by introducing a scalable bias potential into the modeling system. The addition of a bias potential by these three approaches is based on the principle of maximum entropy. This principle implies the addition of a minimal bias to the MD simulation that is capable of bringing the modeled ensemble into agreement with the experiment (Pitera and Chodera, 2012). Biased MD simulations can use both full-atom and simplified (dummy) representations of spin labels (Figure 3A). In addition, in certain cases, such as distances between rigid spin labels, it may be feasible to simplify the system by modeling the unlabeled protein and replacing interspin distances with distances between Cβ or C⍺ atoms (Hays et al., 2019).

ReMD (Figure 3B) performs a user-defined number of simulation replicas for the spin labels, while the rest of the system, including protein and solvent, is modeled only once. This method operates with a global bias potential that is distributed across all modeling replicas (Roux and Islam, 2013; Islam et al., 2013; Islam and Roux, 2015). In contrast, EBMetaD is a single-replica metadynamics-based approach designed to bias a user-defined variable, such as an interatomic distance, by incorporating an adjustable bias potential (Marinelli and Faraldo-Gómez, 2015; Hustedt et al., 2018). BRER simulates multiple replicas of the biased MD to adjust the ensemble distribution of a particular geometric property (e.g., interspin distance) to match the experimentally derived data (Hays et al., 2019). The restrained average dynamics (*RAD*) technique (Figure 3B) also follows the principle of maximum entropy. A key difference between RAD and reMD, EBMetaD or BRER is that MD simulations are directly driven by raw dipolar evolutions rather than distance distributions (Marinelli and Fiorin, 2019). In particular, RAD can model both single and multiple MD simulation replicas.

Choosing a meaningful and effective bias potential, and analyzing biased MD simulations requires experience. Users with less experience in performing MD simulations with non-standard potentials may opt for *unbiased MD methods* instead (Figure 3A).

In principle, *unbiased MD* simulations (Figure 3A), can provide a representation of all protein conformations within the ensemble. Quantification of the individual conformations corresponding to the equilibrium populations in an experiment is difficult to access, especially for slow conformational transitions (Grossfield and Zuckerman, 2009; Sawle and Ghosh, 2016). Similar to biased MD simulations, both full-atom and dummy spin label representations are viable options for unbiased MD simulations (Islam et al., 2013). Unbiased MD simulations require more computational time than biased ones to overcome energy barriers between conformations (Figure 1). To enhance conformational sampling in unbiased MD simulations, multiple replicas starting from different geometries of both the protein and spin labels can be used. Another strategy is to implement different initial velocities in multiple MD replicas (Rice and Brünger, 1994).

To set up a molecular model for conducting biased or unbiased MD simulations, including all-atom or dummy description of spin labels, we recommend the Charmm-GUI module called PDB Manipulator (Jo et al., 2014). In addition, the Charmm-GUI offers many options for constructing and parameterizing membrane proteins such as receptors and transporters in various lipid systems. Lipid mono- and bilayers, nanodiscs, micelles and bicelles, lipid hexagonal phase systems, are available. In each case, the lipid composition is customizable (Feng et al., 2023; Qi et al., 2019; Brown et al., 2024).

### Methods for separate modeling of protein and spin label dynamics

*Discriminating approaches* model protein and spin label conformations independently using different methods. We divide discriminating approaches into static (Figure 3C) and dynamic ones (Figure 3D).

*Static approaches* (Figure 3C) rely on statistical data or physical knowledge to model new variants of molecular geometry and evaluate their reliability (score). Static approaches to modeling spin label conformations work with their full-atom representations during the sampling process. The rotamers of spin labels in the resulting sampled set can be represented by dummy or coarse-grained (CG) models. Static approaches to modeling spin labels include the rotamer library approach (*RLA*) with its modification called *off-rotamer sampling*, and the Monte-Carlo accessible volume (*AV*) sampling (Tessmer et al., 2022). Both RLA and off-rotamer sampling work with a pre-calculated rotamer library, while AV starts with a single spin label structure. In all three approaches, spin label structures are virtually attached to specific labeling sites on the protein structure. The crucial step is the selection of spin label conformations that do not lead to steric clashes (overlapping van der Waals radii) with protein atoms. The main result of all three methods is the ensemble of spin label conformations that can be reliably accommodated at selected label sites. The RLA approach is implemented in the MMM and RosettaEPR programs, and in Python packages such as DEER-PREdict and chiLife (Jeschke, 2018; Alexander et al., 2013; Tesei et al., 2021; Tessmer and Stoll, 2023). AV is implemented in MtsslWizard, mtsslSuite, PRONOX software, and chiLife (Tessmer and Stoll, 2023; Hagelueken et al., 2012; Hagelueken et al., 2013; Hatmal et al., 2012).

Static approaches to modeling protein conformations include statistics-based approaches (neural networks) and a physics-based approach. *Neural networks* for modeling protein conformations, such as AlphaFold3, AlphaFold2, RoseTTAFold2, ESMFold, OmegaFold, and EquiFold are becoming increasingly popular (Jumper et al., 2021; Jeliazkov et al., 2023; Abramson et al., 2024; Baek et al., 2023; Wu et al., 2022). The main reason is that neural networks allow the rapid generation of multiple and diverse conformational models in a short time. However, currently developed neural networks are not necessarily consistent with the laws of physics (Baek and Baker, 2022). Therefore, the results should be further refined using physics-based approaches, such as MD simulations, a dynamic approach that will be considered further, or the static approach implemented in the *Rosetta toolkit* (Leman et al., 2020). Rosetta’s scoring function includes physics-based terms that ensure that the modeled conformations would better reproduce the real geometries. Protein conformations obtained by modeling with Rosetta can be integrated with the RLA approach for modeling spin label conformations described above. Note RosettaDEER, which uses a dummy representation of the rotamer library in its pipeline for efficient modeling of spin-labeled protein conformations in agreement with experimental data (Del Alamo et al., 2021).

*Dynamic discriminating approaches* combine methods that use MD simulations to explore the conformations of either the spin label or the protein (Figure 3D). One such technique for effectively modeling spin label conformations is *CREST/MD*. In a first step, the CREST software samples low-energy conformations of spin labels attached to the specific sites of the protein (Spicher et al., 2020; Pracht et al., 2020). It then performs multiple replicas of short equilibrating MD simulations of the protein labeled with the previously selected lowest energy spin label conformations. In this way, CREST/MD efficiently explores the conformational space of spin labels. Another group of dynamic discriminating approaches performs long MD simulations of an unlabeled protein. To enhance conformational sampling, simplified CG protein models with appropriate modeling parameters (force fields) can be used. In CG models, only a few particles represent each amino acid residue of the protein, which reduces the computational cost but also reduces the accuracy of the calculations (Monticelli et al., 2008). Modern CG force fields such as Martini 3 and SIRAH do not include parameters for spin labels (Souza et al., 2021; Klein et al., 2023). Thus, the simulation frames of the resulting MD trajectories must be supplemented with, for instance, the RLA approach to attach realistic spin label rotamers. Subsequently, spin–spin distances are calculated, and the resulting distributions are compared with the DEER experiment (Wingler et al., 2019).

## Outlook

Distance distributions are the easiest way to integrate sparse EPR data into computational structural biology. Such distributions can be derived from different EPR approaches, in particular CW EPR line-broadening or PDS such as DEER, and compared directly with MD simulations. In this way, MD trajectories can provide us with atomistic structures and dynamics of protein conformations observed in experiment.

There are two major challenges in performing such MD simulations. First, as shown in Figure 1, protein conformations are often separated by high energy barriers, resulting in slow (µs to ms) transitions. This leads to high computational costs and very long simulation times (often weeks or months even on supercomputers), especially for unbiased MD simulations. The second challenge is to obtain reliable simulation parameters (force fields) for the attached spin labels. Force field parameterization is an active area of research with no general solutions yet. Existing tools may not be suitable for a particular task or may be closed source (Jo et al., 2014; Boothroyd et al., 2023). To address the first challenge, we propose to combine MD tools with deep learning approaches. Neural networks can provide a diverse set of protein conformations that serve as starting points for multiple independent and shorter MD replicas. This may improve the efficiency of conformational landscape sampling. The second challenge can be addressed in two ways: either by avoiding the need for parameterization altogether by using discriminating approaches (Figure 3C, D), or by exploring the literature-validated parameters of spin labels. Parameters exist for spin labels such as MTSSL, PROXYL-MTS, BtnRG-TP, Cu^2+^-nitrilotriacetic, and Cu^2+^-iminodiacetic acid (Sezer et al., 2008b; Qi et al., 2020; Bogetti et al., 2020). Alternatively, the parameters for the attached spin labels can be determined independently. In this case, special attention should be paid to the calculation of atomic charges (He et al., 2022). We propose the idea of a database of spin label parameters, compatible with commonly used force fields for proteins, and freely available to the research community.

An alternative method for modeling protein conformations uses DEER distances to guide the modeling process. Here, we refer here to rapidly developing deep learning methods, such as AlphaLink and AFEXplorer, which are capable of incorporating experimentally derived geometric constraints directly into the workflow (Stahl et al., 2023; Xie et al., 2023). Although originally developed for photo-crosslinking mass spectrometry data, AlphaLink can be adapted to DEER distance distributions as descriptors. Such neural networks sample the conformational landscape very efficiently, easily generating hundreds or more models. It is imperative to validate such conformational models using physics-based methods such as MD simulations.

Currently, the only method available to integrate CW EPR data with molecular modeling techniques is RosettaEPR (Alexander et al., 2008). It combines the results of EPR experiments performed on two single cysteine mutants and one double cysteine mutant to calculate interspin distance distributions. The main advantage of this approach is its applicability to physiologically relevant temperatures. Computational approaches that integrate the dynamic information encoded in CW EPR lineshapes with structural biology are still lacking. We suggest that data such as the Heisenberg exchange rate or heuristic accessibility (Altenbach et al., 1989; Altenbach et al., 2005) could be converted to accessible surface area, which then can be used as a sparse descriptor to bias deep learning methods such as AlphaFold3, AlphaFold2, RoseTTAFold2, and ESMFold (Jumper et al., 2021; Jeliazkov et al., 2023; Abramson et al., 2024; Baek et al., 2023). Another possible application of experimental CW EPR data is the filtering and validation of structural models predicted by neural networks or observed in MD simulations (Baek and Baker, 2022).

In summary, we highlight the potential applications of using sparse EPR data for atomistic modeling of protein conformations. We hope that this concise and high-level introduction to the field of integrative modeling using EPR constraints will help interested researchers from other research areas to incorporate these methods into their own research and further advance the field.
